# Optimization of operating conditions for egg setters using computational fluid dynamics simulations

**DOI:** 10.1016/j.psj.2025.105897

**Published:** 2025-09-23

**Authors:** Hee-woong Seok, Rack-woo Kim, Chan-min Kim, Jun-gyu Kim

**Affiliations:** a Department of Smart-Farm Engineering, College of Industrial Sciences, Kongju National University, 54, Daehak-ro, Yesan-eup, Yesan-gun, Chungcheongnam-do, 32439, Republic of Korea; b Animal & Aquaculture Intelligence Research Center, Electronics and Telecommunications Research Institute, 218 Gajeong-ro, Yuseong-gu, Daejeon 34129, Republic of Korea

**Keywords:** Computational fluid dynamics, Egg setter, Setter thermal management, Hatchability enhancement

## Abstract

As the poultry industry continues to expand, improving hatchability during the egg hatching stage is crucial for meeting rising demand in the poultry sector. To address this critical gap, this study used computational fluid dynamics (CFD) to analyze the internal airflow and thermal environment of large-scale setters under various operational conditions, including fan speed, tray-level configuration, and tray turning angle. The objective was to optimize operating conditions to enhance egg hatchability. The results showed that increasing fan speed enhanced internal air velocity, whereas increasing tray levels reduced airflow velocity. Thermal flow analysis demonstrated that maintaining a fan speed of 80 RPM produced optimal temperature uniformity within the setter, ensuring favorable hatching conditions. The study also identified a 15-level tray configuration that was optimal for hatchability, and unidirectional egg turning maintained uniform temperature and humidity distributions. These findings emphasize the importance of balancing airflow, temperature, and humidity to improve hatchability and hatching productivity. In conclusion, this study provides valuable insights into optimizing large-scale setters for improved poultry production efficiency and sustainability by proposing effective operational strategies to enhance hatchability.

## Introduction

### Background

Population growth and climate change have an increasingly severe impact on food security worldwide. As the global population rises, the demand for food continues to surge; furthermore, and climate change poses significant challenges to traditional agricultural production methods (FAO, 2020). Poultry holds a key position in the food supply chain because of its relatively low production costs and rapid production rates compared to other sources of animal protein (OECD/FAO, 2024).

The scale and economic importance of the poultry industry continue to expand; consequently, the global demand for poultry products is steadily increasing. As of 2025, broiler meat production is expected to reach 632,000 tons, and the global broiler population is projected to increase to approximately 99.45 million birds (World Bank, 2023; Global Poultry Market Insights, 2024). These trends underscore the critical role of the poultry industry in ensuring food security and suggest that its importance and scale will continue to grow. To meet the demands of the continuous expansion of the poultry industry, it is crucial to enhance the hatchability rate during the hatching stage of egg incubation, which is a key phase in poultry breeding (Boleli, 2016). Currently, two methods of egg incubation are used: natural incubation with hens and artificial incubation. The former involves brooding by the mother hen; notably, the hen does not lay new eggs while raising hatched chicks, making it economically less advantageous than artificial incubation. Consequently, natural incubation is used less frequently, and the development of artificial incubation techniques using setter technology has garnered significant attention. Artificial incubation, now aptly referred to as artificial hatching, involves the artificial incubation of fertilized eggs by controlling the temperature, humidity, and ventilation within the facility. This method yields higher egg laying and lower mortality rates compared to natural incubation, thus maintaining stable production levels. Recently, large setters equipped with advanced control devices have been developed and distributed.

However, setters simulating hatching environments can cause mass mortality of embryos if developmental environments are not controlled within the facility. This, coupled with the occurrence of diseases caused by bacterial infiltration, can lead to sudden decreases in hatchability. The internal temperature of the setter significantly affects the hatchability rate and growth of the chicks after hatching. Given that embryos within eggs are ectothermic and cannot regulate their body temperature (Piestun et al., 2008; Walter and Seebacher, 2009), maintaining the internal temperature of the setter at an optimal hatching temperature is the most crucial factor influencing setter performance (Lourens et al., 2005, 2006). The optimal hatching temperature for eggs ranges from 37.2 °C and 37.8 °C, and maintaining temperatures during embryonic development is critical, as temperatures above or below the optimal range can lead to a rapid decrease in hatchability rates. Therefore, to enhance egg productivity and increase hatchability rates, it is necessary to appropriately control developmental environmental factors, such as airflow, temperature, humidity, and CO2 levels, within the setter.

However, maintaining a uniform internal temperature becomes more challenging as setter size increases (Wilson, 1991; Decuypere, 1994). Depending on the position of the eggs, the speed of air circulation inside the setter, the weight of the eggs, and their age, temperature differences between the eggshell and the air within the setter can range from 0.4–4.0 °C, leading to unevenness (Lourens et al., 2005; Joseph et al., 2006; Elibol and Brake, 2008). A temperature deviation of over 1.1 °C inside the setter can impair the growth, development, and hatching viability of embryos, especially reducing the ability of chicks to maintain their body temperature after hatching under cold stress conditions (Romijn and Lokhorst, 1956). Thus, although the operation of the internal air-conditioning system in the setter is regulated by controlling internal fans for ventilation, maintaining an appropriate temperature is challenging. The internal environment may vary depending on the arrangement of eggs and the angle at which they are turned. Previous studies have primarily focused on small-scale setters or setters developed outside the country, with limited research on large-scale setters. Therefore, there is a need to optimize the operation of large-scale setters.

### Literature reviews

Previous studies aimed at evaluating the internal environment of setters and identifying the optimal operational conditions have been conducted using field experiments and simulations. However, most studies have focused on small-scale setters or setters developed outside the country, with relatively limited analyses of large-scale setters (Elibol and Brake, 2006; Abiola et al., 2008; Özcan et al., 2010; Sözcü et al., 2021; Niranjan et al., 2021; Kommey et al., 2022). These studies on small-scale setters were conducted in environments where factors such as air circulation, thermal flow, and humidity were relatively uniform within a limited space, and thus do not adequately account for the complex environmental variables that arise in large-scale setters.

Large-scale setters face numerous environmental challenges, including uneven temperature distribution, airflow imbalance, and heat loss. In large setters, maintaining internal temperature uniformity is challenging, and imbalances in air circulation and heat flow significantly affects hatchability. As observed in the cases of failure reported by setter manufacturers, such issues can lead to decreased hatchability, delayed chick growth, and disrupted embryo development. Therefore, comprehensive analysis and optimization of large-scale setters are essential.

Although previous studies have focused on improving the efficiency of small-scale setters, studies aimed at efficient operation and resolving issues within large-scale setters is lacking. To optimize the internal environment of large-scale setters, it is necessary to extend the findings of small-scale setter studies to large-scale systems and conduct comprehensive research that reflects the practical requirements of industrial operations.

### Research scope and goals

To address this critical gap, this study aimed to develop an optimal operation strategy for setters using computational fluid dynamics (CFD) to analyze the internal thermal environment of large setters according to their operating conditions. CFD enables the prediction and visualization of the aerodynamic flow of fluids by interpreting physical laws, specifically airflow simulations. Using CFD, this study analyzed the internal airflow and thermal environment by designing setters. Additionally, by selecting cases based on various operating conditions and performing simulation analyses, this study sought to derive an optimal operating strategy for a heating, ventilation, and air conditioning (HVAC) system for setters, with the goal of enhancing the hatching rate.

## Materials and methods

**Fig. 1:**
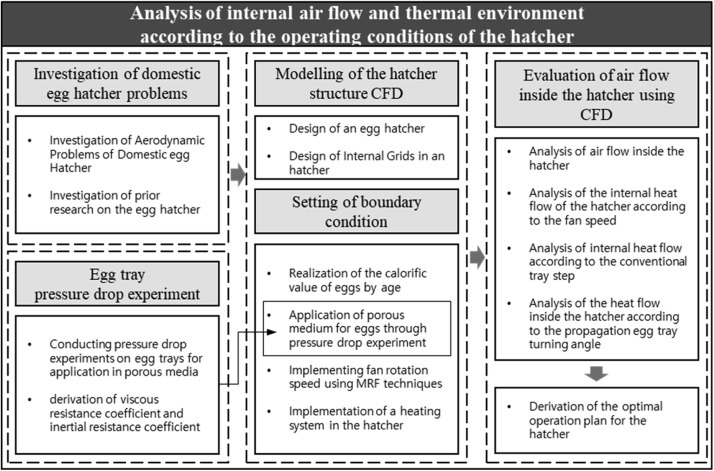
Flow chart for deriving the optimal operating conditions for a setter.

CFD was used to design a setter to analyze the internal air and thermal flow. Various operational conditions were selected for the simulation analyses. This study aimed to establish optimal operational strategies for the HVAC system of a setter to enhance egg hatchability. To achieve this, typical aerodynamic issues that occur in setters were identified through field surveys and literature reviews. Moreover, a validated simulation model was employed for the CFD simulations and subsequent modeling. Using the designed CFD simulation model, analyses of the air and thermal flows within the setter were performed, considering variables such as fan rotational speed, number of trays, and angle of egg turning (Fig. 1).

### Target facility

**Fig. 2:**
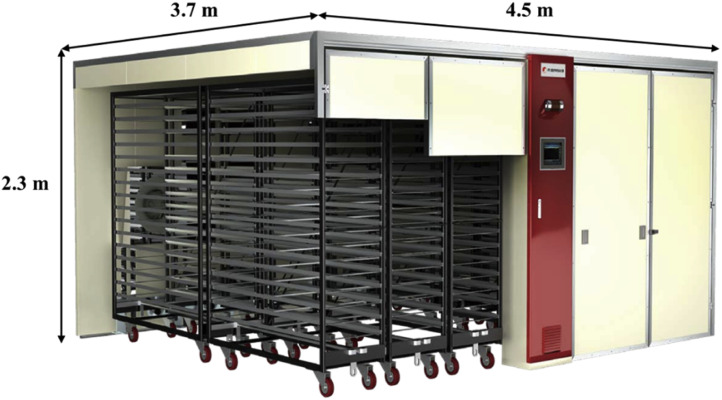
Schematic diagram of the egg setter.

The target facility was a KHS-576 setter from Daesang Kiwoomi System Co., Ltd. (Gangneung-si, Gangwon-do, South Korea); the shape and specifications of the setter are shown in Fig. 2 and Table 1. The KHS-576 setter is 3.7 m in width, 4.5 m in length, and 2.3 m in height, with an insulation thickness of 0.25 m. The setter contains 16 trays capable of holding 150 eggs each, which are loaded onto a single trolley. A total of 24 trolleys were arranged, allowing for the incubation of 57,600 eggs.

**Table 1:** Specifications of the egg setter.

Mode		KHS-576
Egg Setter	Width (m)	3.7
	Length (m)	4.5
	Height (m)	2.3
Tray	Width (m)	0.5
	Length (m)	1.5
	Height (m)	0.03
	level	16
	Number of eggs	150
Inclination	Angle (°)	0, 45, −45
	Cycles (hr^−1^)	1
Fan	Quantity	2
	Speed (RPM)	80

**Fig. 3:**
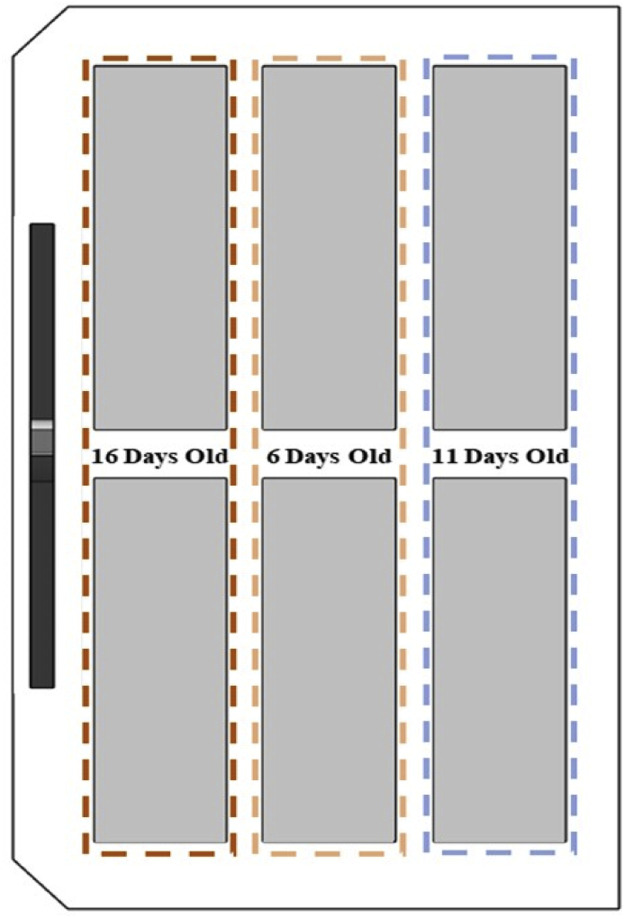
Schematic diagram of egg tray arrangement by age in the egg setter.

Given the differing heat outputs of eggs at various developmental stages, Daesang Kiwoomi System Co., Ltd. implemented a strategic arrangement of trolleys within the incubator to enhance internal temperature uniformity, as depicted in Fig. 3. The trolleys bearing 16-day-old eggs, which had the highest heat output, were positioned closest to the fans. Conversely, trolleys with 11-day-old eggs, which exhibited the second highest heat output, were placed farthest from the fan. Trolleys carrying 6-day-old eggs were strategically located between these two, facilitating the operation of a large incubator to ensure an even internal temperature conducive to egg development.

**Fig. 4:**
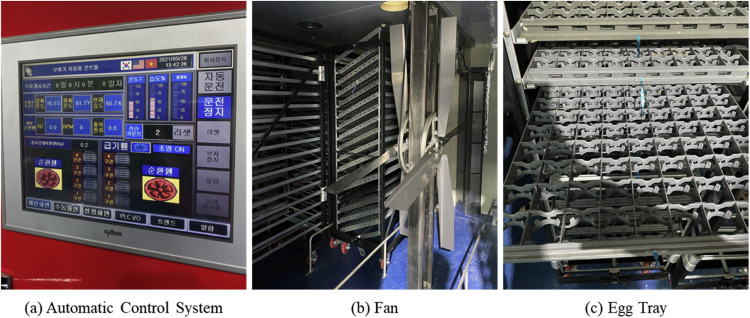
Components of the egg setter system.

Additionally, as illustrated in Fig. 4, the setter was equipped with two heat radiators to maintain optimal hatching temperatures for the eggs and fans at the center for air circulation.

To prevent the embryos from adhering to the eggshell membrane, an egg-turning device was installed to alter the inclination of the trays by 45° to the left and right every hour, thereby facilitating egg rotation.

### Computational fluid dynamics

CFD simulations, which numerically solve the partial differential equations governing fluid flow phenomena, have been used extensively across a broad spectrum of engineering disciplines, including mechanical, environmental, chemical, aeronautical, and aerospace engineering. CFD simulations offer economic advantages over field experiments while providing flexibility in controlling variables. Hence, they are widely employed for various applications such as thermal flow evaluation and ventilation analysis.

A CFD simulation technique based on the finite volume method was used to compute Reynolds numbers using the Navier–Stokes equations for each cell within and outside the hatcher region. The main module used for the computations, as listed in Table 2, was Fluent (version 23.0; ANSYS Inc., Canonsburg, PA, USA). Additionally, the simulation treats the flow as incompressible and turbulent across the entire domain. For accurate prediction of fluid flow and heat transfer, the Realizable k-ε model was used, allowing appropriate adjustment of wall surface meshes (Liu et al, 2012).

**Table 2:** Design conditions for the computational fluid dynamics simulation models.

Parameter	Model setting
Solver	Pressure-based
	Implicit formulation
	Steady-state analysis
	3D simulation
Turbulence Model	Realizable k-ε model

### Egg tray pressure drop experiment

**Fig. 5:**
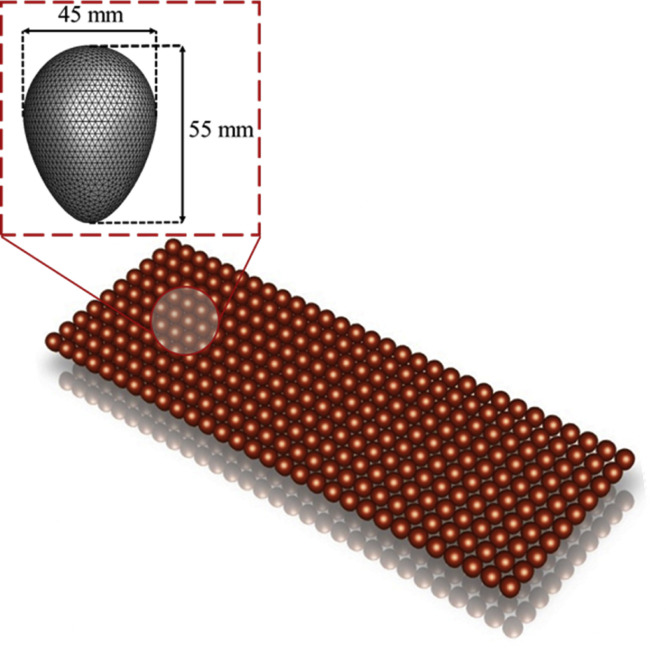
Implementation of an egg in the computational fluid dynamics simulation for applying porous media to eggs.

**Fig. 6:**
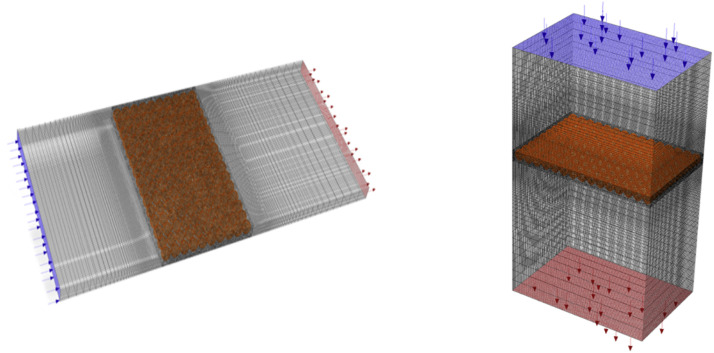
X, Z-axis (left) and Y-axis (right) pressure drop modeling.

To perform flow analysis, complex geometries such as eggs and trays can be simplified as porous media in CFD simulations by considering various factors, including grid quality and computation time (Ju et al., 2018). Accordingly, as illustrated in Fig. 5, the eggs were assumed to be elliptical, with the longest circumference of 5.2 cm and one pointed end, based on the average size of the eggs. To analyze the airflow around the trays holding the eggs as porous media, an experiment was conducted to determine the pressure drop across the egg trays (Fig. 6).

In a large hatcher, the airflow encounters pressure drops due to flow resistance as it passes through the trays holding the eggs. A superficial velocity porous formulation was used for analysis. The porous media were modeled by adding a term representing momentum to the standard fluid flow equations, as expressed in Equations (1) and (2).

(1) $$S_{i}=-\left(\sum_{j=1}^{3}D_{\text{ij}}\mu \nu_{j}+\sum_{j=1}^{3}C_{\text{ij}}\frac{1}{2}\rho |\nu |\nu_{j}\right)$$

(2) $$S_{i}=-\left(\frac{\mu }{\alpha }\nu_{i}+C_{2}\frac{1}{2}\rho |\nu |\nu_{i}\right),$$

where S_i_ represents the source term for the momentum equation, i the magnitude of velocity, α the permeability of the porous media, and C_2_ the inertial resistance coefficient. Momentum contributes to pressure within the porous medium, generating a pressure drop proportional to the fluid velocity within the cell.

To model egg trays as a porous medium, CFD was used to calculate the pressure drop across the egg trays as a function of wind speed. The viscous and inertial resistance coefficients were derived from the relationship between wind speed and pressure drop.

### Computational fluid dynamics simulation model design

**Fig. 7:**
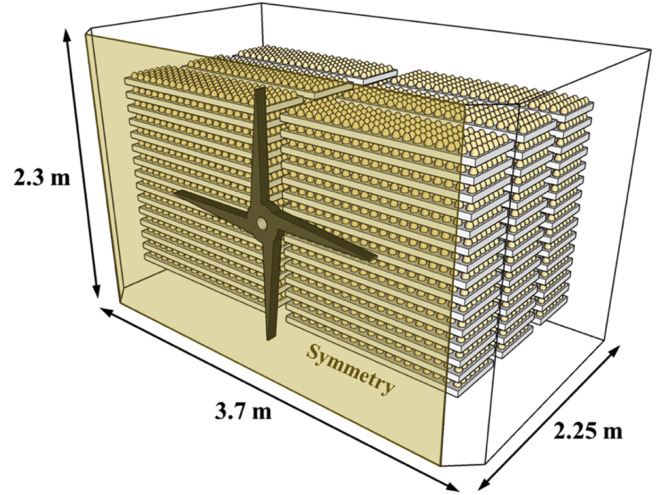
Design of the egg setter based on computational fluid dynamics simulation.

The setter geometry was designed based on the blueprints of the KHS-576 setter, as shown in Fig. 7. Considering that the target facility has a symmetrical form on the left and right sides, only half of the original shape was 3D-modeled to enhance computational efficiency and convergence. Instead of replicating the exact shape of the eggs, they were designed as rectangular shapes and modeled as porous media. To model eggs as a porous medium in the CFD simulation, the drag coefficient was calculated through a pressure drop experiment, which was then used as a boundary condition.

For the two circulation fans located at the center of the setter, modeling was conducted according to the design blueprints, and a moving reference frame (MRF) model was employed to account for the fans. This approach used a rotating coordinate system to simulate fan rotation by rotating the surrounding grid. The MRF model is an analytical technique used in CFD to model problems involving rotating components. The fundamental principle of the MRF model is to establish an MRF zone within a small volumetric area of mesh cells created around the rotator, thereby allowing the simulation of fluid flow around the MRF zone while the rotator remains stationary. During the simulation process, the rotation speed and axis of the rotating region were considered, enabling accurate prediction of fluid flow. The rotator was treated as stationary to simplify the simulation and maintain a stable flow field relative to the geometric structure (Fernandes et al, 2023).

In addition, approximately 6,000,000 mesh elements were generated across the computational domain, with a final mesh size of 1.0 × 10^−2^m The average skewness of the mesh was 0.07, and the average orthogonal quality was 0.89. These values fall within the ranges considered suitable for CFD simulations, with skewness < 0.5 and orthogonal quality > 0.7.

### Computational fluid dynamics simulation boundary conditions

**Fig. 8:**
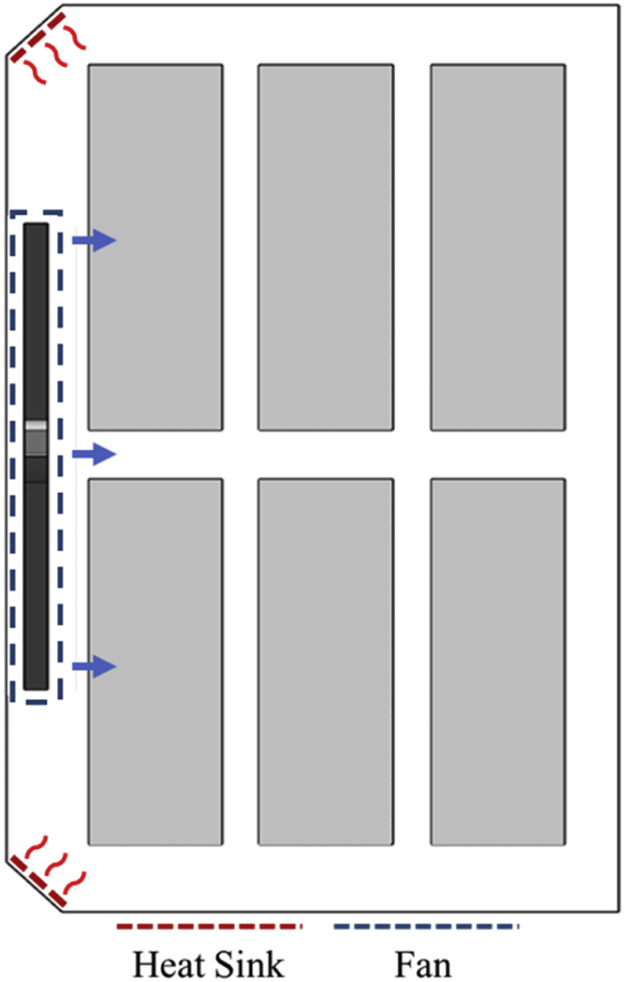
Schematic diagram of boundary conditions for the computational fluid dynamics simulation of the egg setter.

To analyze the CFD simulation model, it is essential to specify the boundary conditions, material properties, and parameters for numerical calculations. The boundary conditions of the simulation model used in this study are presented in Fig. 8 and Table 3. Owing to the variation in the heat output of eggs at different stages of development, the average value of egg heat production was calculated through a review of prior research and was used as a boundary condition for the CFD simulation. The heat output was modeled as –5.4 W m^−3^ for 6-day-old eggs, 1.8 W m^−3^ for 11-day-old eggs, and 19.6 W m^−3^ for 16-day-old eggs (Romijn and Lokhorst, 1956). For the heat radiators that maintain appropriate temperatures inside the setter, the boundary condition was set to emit heat at 300 W m^−2^, based on the performance of products used in actual large setters, and modeled as Heat Flux in the CFD simulation. Additionally, considering the enclosed nature of the setter interior, the heat transfer coefficient was set at 30 W m^−2^ K^−1^, with an external temperature of 20 °C and wall thickness of 0.5 m, to model heat loss through the walls.

**Table 3:** Boundary conditions set for the computational fluid dynamics simulation to determine the optimal operating conditions for the setter.

Type			Values
Egg	Heat flux (W m^−3^)	6-days-old	−5.4
		11-days-old	1.8
		16-days-old	19.6
Heat sink	Heat flux (W m^−2^)		300
Wall heat loss	Heat transfer coefficient (W m^−2^ K^−1^)		30
	Free stream temperature (°C)		20
	Wall thickness (m)		0.5
Humidity (%)			70

To accurately model the humidity conditions within the setter, a species transport model was used in CFD Fluent to reflect the dynamic behavior of water vapor inside the setter. The actual setter system maintains a consistent humidity of 70 % through automatic humidification, which is activated when the humidity drops below 70 % and stops when it reaches the target value. This behavior was replicated by setting the initial internal humidity to 70 % using the patch setting in the CFD model. The species transport model enabled the simulation of moisture movement and its interaction with airflows, ensuring that the internal humidity remained at 70 %, while also allowing for variations in local humidity due to changes in temperature and airflow patterns.

This approach ensured that the simulation accurately represented the real-world dynamics of the hatching environment, where internal humidity fluctuates in response to temperature and fan speed variations. Using the patch feature, the simulation commenced at a baseline humidity of 70 %, which was dynamically regulated throughout the simulation process to mirror the operational behavior of the actual setter. This enabled a comprehensive analysis of the effects of varying fan speeds, tray configurations, and other operational factors on the distribution and stability of humidity inside the egg setter.

### Computational fluid dynamics simulation case study

The CFD simulation cases in this study are detailed in Table 4, comprising 45 cases across five different egg tray turning angles, three different egg tray configurations (14, 15, and 16 levels) and three fan speeds. For the egg tray angles, the basic conditions were set to 0°, 45°, and −45°, which are the actual operational angles used by Daesang Growmee System Co., Ltd. Additionally, conditions using egg tray turning angles of 45°, −45°, and 45°, as well as −45°, 45°, and −45°, which are used internationally, were included, comprising a total of five conditions. Although setters are typically operated in 16 levels, considering the lower hatchability rates, 14- and 15- level conditions were also considered to analyze the effect of tray count on internal temperature. Although the standard operational fan speed was 100 RPM, speeds of 80 RPM and 120 RPM were also explored. Fan speed, tray- level configuration, and turning angle were specifically selected because they are the primary operator-adjustable factors that directly influence airflow, temperature, and humidity in commercial setters. Other structural variables (e.g., humidification nozzle or heater layout/capacity) were held constant to reflect the manufacturer’s as-built design, thereby isolating the effects of operating conditions.

**Table 4:** Computational fluid dynamics simulation cases for determining the optimal operating conditions for the setter.

Conditions			Number of cases
Fan	Speed (RPM)	80	3
		100	
		120	
Tray	level	14	3
		15	
		16	
	Angle (${}^{\circ }$)	0, 0, 0	5
		45, 45, 45	
		−45, −45, −45	
		45, −45, 45	
		−45, 45, −45	
Total cases			3 × 3 × 5 = 45

**Fig. 9:**
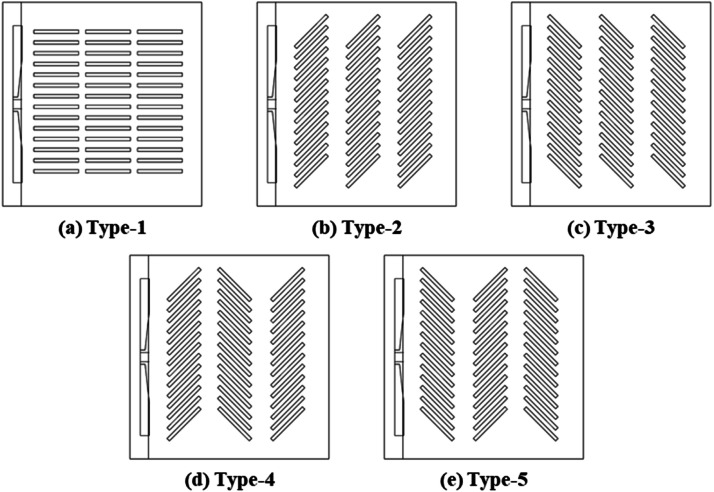
Configuration for analyzing airflow inside the setter based on the turning angle of egg trays.

Additionally, to analyze the efficiency of the simulation results, the types were designated based on the turning angle of the egg tray, as shown in Fig. 9. An egg tray turning angle of 0° was categorized as Type-1, 45° as Type-2, and −45° as Type-3, and sequences of 45°, −45°, and 45° were defined as Type-4 and of −45°, 45°, and −45° were defined as Type-5.

### Computational fluid dynamics simulation analysis method

**Fig. 10:**
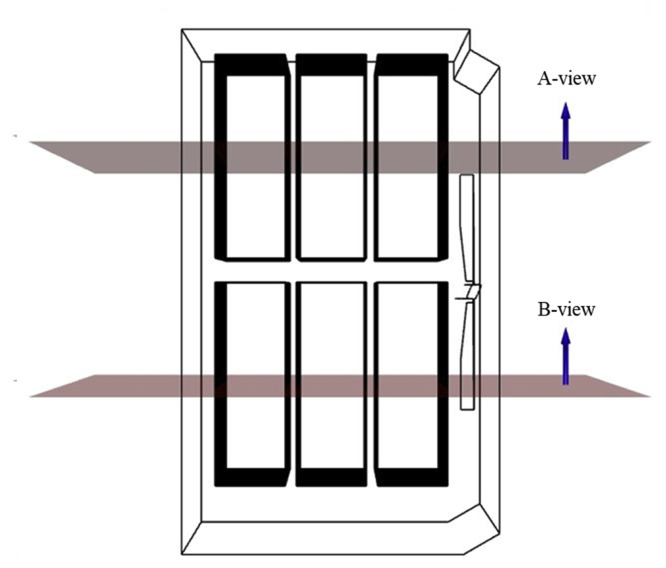
Selection of A-view and B-view positions for evaluating the thermal environment inside the setter.

To evaluate the airflow and thermal characteristics inside the egg setter under various operating conditions, the internal spatial domain was analyzed using a standardized sectional approach. A reference X–Y plane was defined at the horizontal center of the egg tray trolley, where the eggs were positioned during incubation (Fig. 10). This plane was further divided into two observation regions, A-view and B-view, based on the front and rear sections of the tray trolley center, rather than its outer boundaries. This segmentation enabled a consistent comparison of airflow vectors and temperature distributions across all simulation cases, including variations in fan speed, tray-level configuration, and egg tray turning angle.

The A-view corresponds to the front side of the tray trolley, and the B-view corresponds to its rear side, enabling the assessment of potential asymmetries and flow imbalances induced by structural and thermal effects within the core zone of the incubator. These views were used as the primary surfaces for extracting the simulation data and assessing the spatial uniformity of temperature and airflow.

### Computational fluid dynamics simulation model validation

**Fig. 11:**
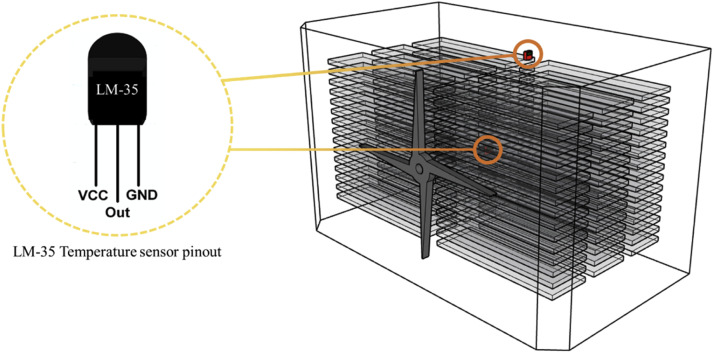
Sensor placement within the egg setter.

The CFD simulation model was validated by comparing the simulation results with temperature sensor measurements inside the KHS-576 setter. The validation simulation was conducted under actual hatching conditions with a fan speed of 100 RPM and a tray configuration of 16 levels, representing the typical operating settings of the KHS-576 setter. For the temperature measurements, a sensor module using LM-35 temperature sensors was configured, and the data collected from this module were used for validation. As shown in Fig. 11, Sensor-1 was located 0.11 m from the upper wall at the position of the 11-day-old egg tray, and Sensor-2 was placed at the center of the setter. Temperature data were collected over a 24-hour period at 5-minute intervals and aggregated into hourly averages for comparison. The error rates between these averaged measurements and the simulation results were calculated to evaluate model accuracy.

Considering that the setter operated under steady thermal conditions in an enclosed space with minimal external interference, a one-day measurement was deemed sufficient to capture the representative thermal behavior. The simulation model was validated against these measurements, confirming the reliability of the predicted temperature distribution.

## Results and discussion

### Calculation of pressure drop across the egg tray for porous media modeling

**Fig. 12:**
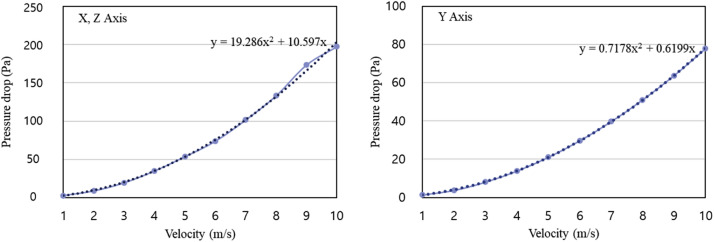
Egg pressure drop test results and approximation formulae.

The viscous and inertial resistance coefficients applied to egg trays were derived numerically by defining two-directional vectors in a three-dimensional space environment for each direction. Table 5 presents the results of the pressure drop calculated across the egg trays as a function on the flow rate, showing that the pressure drop increased proportionally with the mass flow rate at the tray inlet. Using the analysis results of the pressure drop with respect to the mass flow rate of the egg trays, an empirical formula was derived, as illustrated in Fig. 12, by fitting a trend line passing through the points to generate the equation. This empirical formula was then used to derive the viscous and inertial resistance coefficients for the porous medium, which were applied as Porous Media values in the CFD simulation, as listed in Table 6.

**Table 5:** Results of the pressure drop tests on eggs in the computational fluid dynamics simulation.

Velocity (m s^−1^)	Pressure	
	X, Z	Y
1	3.29	1.22
2	11.39	4.30
3	24.52	9.28
4	43.24	16.02
5	63.82	24.54
6	90.95	34.79
7	122.19	46.75
8	158.49	60.37
9	198.68	75.81
10	245.05	92.94

**Table 6:** Parameters for porous media of an egg tray in the computational fluid dynamics simulation.

Porous Media		
Axial direction	C^2^	$\frac{1}{\alpha }$
X, Z	43.168 m^−1^	811913.462 m^−1^
Y	18.963 m^−1^	560564.677 m^−1^

### Computational fluid dynamics simulation validation

***Validation of Geometric Representation Uncertainty.*** The accuracy of CFD simulations relies not only on the boundary conditions and numerical methods, but also on how precisely the geometry of the target system is represented. In this study, a three-dimensional model of an egg setter was constructed based on actual technical drawings to ensure high fidelity in the geometric representation. Particularly, the internal fan was modeled with its full geometry to account for the rotational flow characteristics in the simulation.

For egg trays, fully resolving the detailed geometry would result in excessive computational costs owing to their structural complexity and repetitiveness. Therefore, the trays were simplified by using a porous medium approach. This simplification was based on experimentally measured pressure drop data, allowing the model to reflect the flow resistance effect of densely arranged eggs in a numerically efficient manner.

This approach enabled the CFD model to maintain high geometric accuracy while ensuring computational efficiency, while effectively minimizing the uncertainty in the geometric representation.

***Verification of Mesh Quality and Grid Resolution***. The convergence and reliability of the CFD results are strongly influenced by the quality and configuration of the computational mesh. In this study, a systematic procedure was employed to minimize numerical uncertainties arising from the mesh design stage, thereby ensuring numerical stability and solution accuracy. Based on the validated geometric model, a three-dimensional mesh was generated and its quality was assessed using two widely accepted indicators skewness and orthogonal quality as detailed in the Materials and Methods. These checks confirmed that the mesh satisfied commonly accepted thresholds for CFD applications. The average simulation time per case was approximately 100 min, which was well within the limits of the available computational resources. Given these conditions, a formal grid-independence test was omitted because the mesh was deemed sufficiently fine and stable for the intended analysis. Thus, the adopted mesh design methodology is considered to provide an appropriate balance between computational efficiency and numerical accuracy, with minimal uncertainty introduced by the grid configuration.

***Assessment of Boundary Condition Uncertainty.*** The accuracy of CFD simulations is highly sensitive to the specifications of the boundary conditions because poorly defined conditions can become a major source of numerical uncertainty. To address this, the present study verified the reliability of key boundary conditions, specifically ventilation and thermal energy inputs, based on a combination of field measurements and existing literature.

For the ventilation boundary condition, the geometry and operating characteristics of the fan were modeled using field-measured specifications. The internal circulation fan of the egg setter was constructed in three dimensions to match its actual shape, and the simulation incorporated both induced airflow and rotational effects. This ensured that the volumetric flow rate and airflow behavior within the chamber were realistically reproduced.

With respect to the thermal boundary condition, the metabolic heat generation from the fertilized eggs was implemented as a source term, based on physiologically validated values reported in the literature. For the heating system, the heat input was defined as a surface heat flux condition using specifications such as the heat transfer mode and operating temperature range provided in the manufacturer's technical documentation. The location and surface area of the heater were precisely modeled based on the design schematics.

The physical properties of the outer wall, such as insulation material, thickness, and thermal conductivity, were also incorporated into the model. This allowed for a numerical representation of heat loss through the enclosure. Collectively, these efforts minimized the numerical uncertainties related to the thermal and fluid flow boundary conditions in the CFD model.

***Validation of Steady-State Convergence Assumption.*** In this study, a steady-state CFD approach was employed to analyze the thermal and airflow characteristics within the egg setters. The internal space of the setter is structurally enclosed. During operation, external environmental variations are minimal, while the internal conditions remain relatively stable. Even with an increased number of trays, the system primarily experiences a higher spatial density of heat sources than rapid or time-dependent fluctuations in the thermal loads. Therefore, the necessity for a transient simulation was deemed low for this application.

Importantly, the objective of this study was not to track the dynamic behavior of the system over time, but to obtain steady-state distributions of temperature and airflow under defined operating conditions. Hence, the key criterion for evaluating the validity of the numerical model was not its temporal resolution, but rather the accuracy and reliability of the steady-state solution.

Steady-state simulations efficiently capture representative thermal and fluid flow patterns while reducing computational costs. Given the characteristics of the system and analytical goals of this study, a steady-state modeling framework was considered appropriate and justified.

**Fig. 13:**
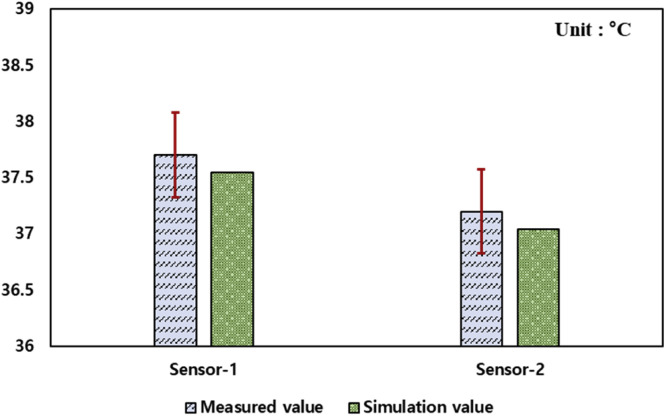
Comparison of computational fluid dynamics simulation measurements conducted at the same location as the actual sensor measurements.

***Final Validation against Experimental Data.*** Fig. 13 shows a comparison of the temperature values measured by the internal temperature sensors of the setter and those obtained from the CFD simulations. For the validation case, simulations were conducted under actual operating conditions in the field using an egg tray with 16 levels and a fan speed of 80 RPM. The average values of the internal temperature sensor measurements in the setter were used, whereas the CFD simulation temperature values were obtained by creating measurement points at the positions of the actual temperature sensors installed inside the setter. The average temperature of Sensor-1 was 37.7 °C, and that of Sensor-2 was 37.2 °C. In the CFD simulations, the temperature of Sensor-1 was calculated as 37.55 °C, and that of Sensor-2 was 37.04 °C. The error rate between the internal temperature sensor measurements of the setter and the point measurements of the CFD simulation at the sensor positions was less than 1.0 %. This indicates that the CFD simulation model is highly accurate and can serve as a viable model under various setter operating conditions.

### Analysis of internal airflow within the setter

**Fig. 14:**
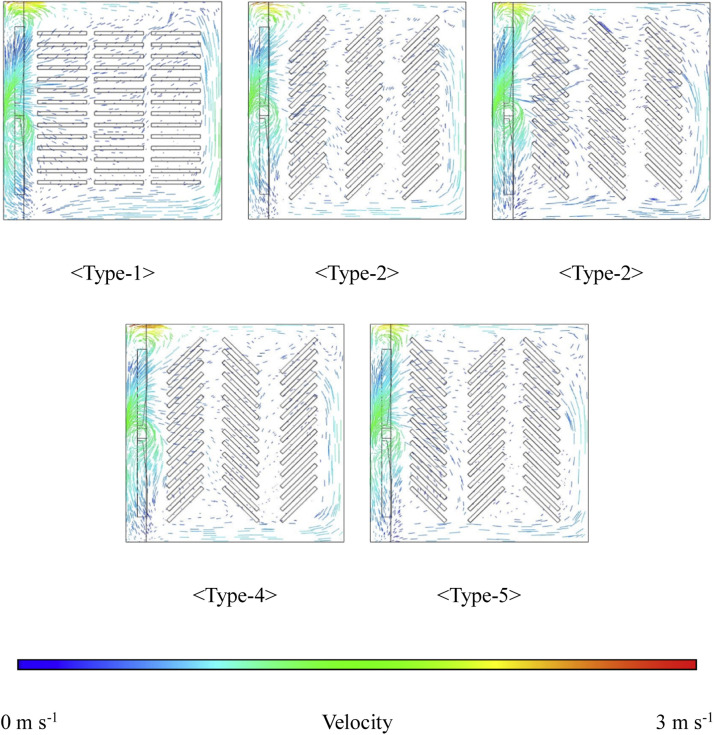
Analysis of the air vector field inside the setter according to tray angle (based on 14 tray levels, 80 RPM).

**Fig. 15:**
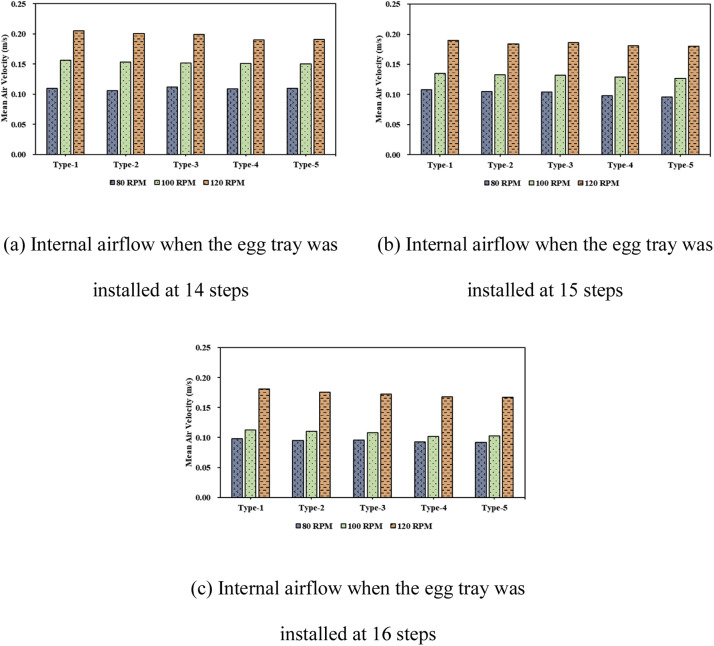
Analysis of internal airflow according to fan speed.

The results of the airflow distribution analysis within the setter based on the operational conditions (fan speed, number of tray levels, and egg tray turning angle) are shown in Figs. 14 and 15. Fig. 15 shows the airflow for the turning-angle cases at 14 tray levels and a fan speed of 80 RPM. For Type-1, air was observed to circulate well between the trays, and for Types-2 and -3, the air circulation was better than that of Types-4 and -5. This indicates that in the case of Type-1, where egg trays were aligned straight, the flow resistance across the trays was lower, leading to better air circulation within the setter. As the egg tray turning angles were tilted to 45° and −45°, the flow resistance to airflow increased, resulting in less efficient air circulation inside the setter.

Furthermore, regardless of the number of tray levels, an increase in fan speed resulted in a higher average air velocity throughout the setter. Conversely, as the number of tray levels increased, the velocity between the trays decreased. Specifically, when the egg tray levels were 14, 15, and 16, the average velocities between the trays were 0.11, 0.108, and 0.098 m s−1, respectively, indicating a decreasing trend. For the 14- level egg tray configuration, the increase in velocity between the trays was most significant with an increase in fan speed; however, this effect diminished as the number of tray level count increased. This suggests that with an increase in the egg tray level count, even though the fan speed increased, the internal air resistance also increased, leading to a reduced increase in the velocity between trays.

As the fan speed increased, the air velocity inside the setter increases, whereas increasing the number of tray levels decreased the internal air velocity of the setter. While appropriately adjusting the airflow is crucial for maintaining optimal hatching temperatures for eggs, it is also necessary to analyze the internal thermal flow to derive optimal operating conditions (appropriate fan speed and number of tray levels).

### Analysis of internal thermal distribution within the setter

**Fig. 16:**
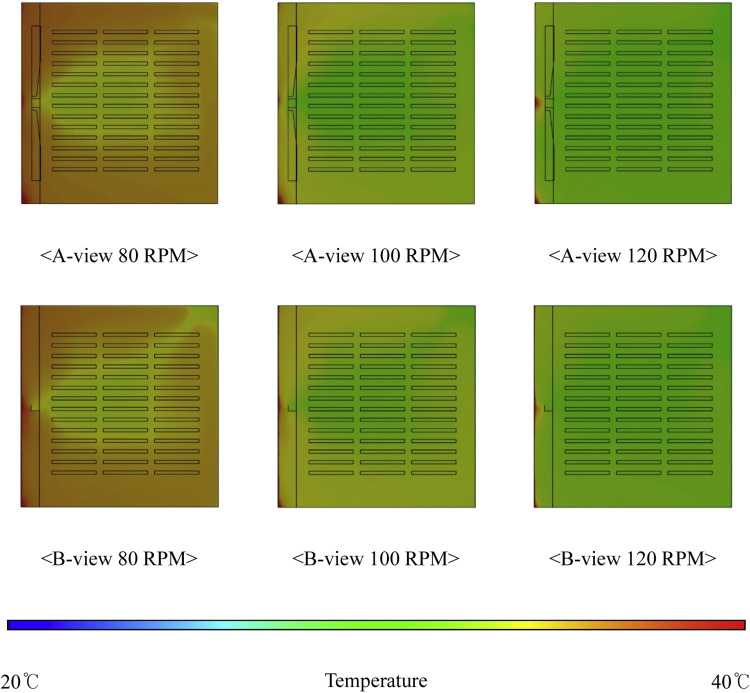
Contours of the internal temperature of the egg setter according to fan speed (based on 14 tray levels, 0° tray angle).

**Fig. 17:**
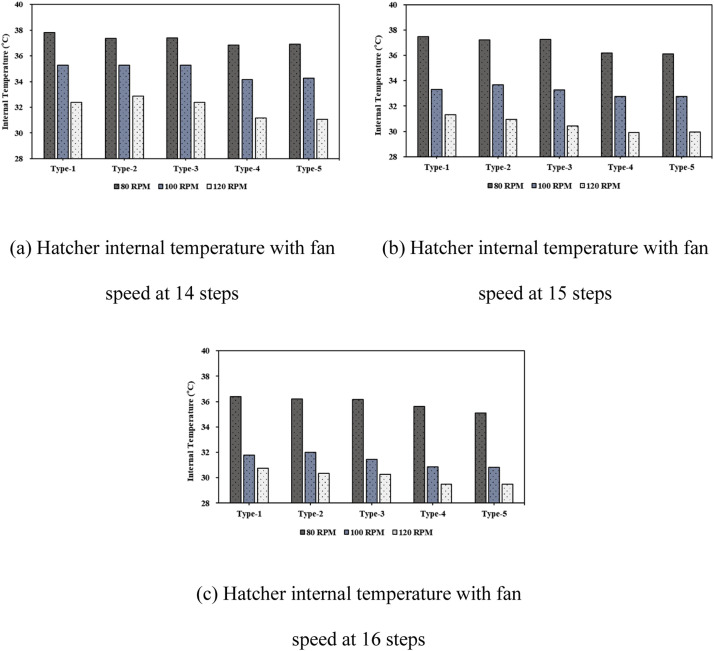
Internal temperature in the setter according to the number of egg tray levels.

***Analysis of Internal Thermal Distribution within the Hatcher based on Fan Speed.*** The results of the thermal-flow analysis influenced by fan rotation speed within the setter are shown in Fig. 16. This figure depicts the temperature contours observed as fan speed increased, with 14 egg tray levels and an egg-tray turning angle of 0°. At a fan speed of 80 RPM, the average temperature inside the setter was 37.81 °C, which decreased to 35.27 °C at 100 RPM and further to 32.38 °C at 120 RPM. When the number of egg tray levels was increased to 16, the temperature at 80 RPM was 37.12 °C, and at 120 RPM it further reduced to 31.17 °C, showing a decrease of approximately 5.66 °C. With an increase in the number of egg tray levels from 14 to 16, the extent of the reduction in internal temperature caused by higher fan speed increased by approximately 0.23 °C (Fig. 17, Table 7). An increase in the fan speed led to a decrease in the internal temperature of the setter, resulting in a more uneven temperature distribution. This phenomenon can be attributed to the sealed environment of the setter, with the boundary conditions set to allow heat loss through the walls. Consequently, as the fan speed increased, the warmer air inside the setter lost heat to the cooler outer walls, leading to a decrease in the internal temperature and a more uneven temperature distribution.

**Table 7:** Results of the heat flow analysis inside the egg setter (°C).

Type	14 levels			15 levels			16 levels		
	80 RPM	100 RPM	120 RPM	80 RPM	100 RPM	120 RPM	80 RPM	100 RPM	120 RPM
1	37.81	35.27	32.38	37.46	33.29	31.30	37.12	32.28	31.17
2	37.37	35.26	32.87	37.22	33.68	30.95	36.70	32.48	30.64
3	37.40	35.28	32.37	37.24	33.27	30.44	36.69	31.93	30.12
4	36.83	34.17	31.15	36.18	32.75	29.92	36.10	31.34	29.86
5	36.90	34.28	31.07	36.11	32.77	29.94	35.59	31.33	29.88

Maintaining an optimal hatching temperature is critical for embryo development. In the present study, the optimal hatching temperature for eggs was maintained consistently within the desired range of 37.2 °C to 37.8 °C by adjusting the fan speed and ensuring internal thermal uniformity.

Finally, when the fan speed within the setter was maintained at 80 RPM, temperature uniformity was achieved and the optimal hatching temperature was maintained. To maintain the internal temperature of the setter at the optimal hatching temperature, adjusting the fan speed according to setter size is more effective than simply increasing the fan speed.

### Analysis of internal thermal flow within the hatcher according to the number of tray levels

**Fig. 18:**
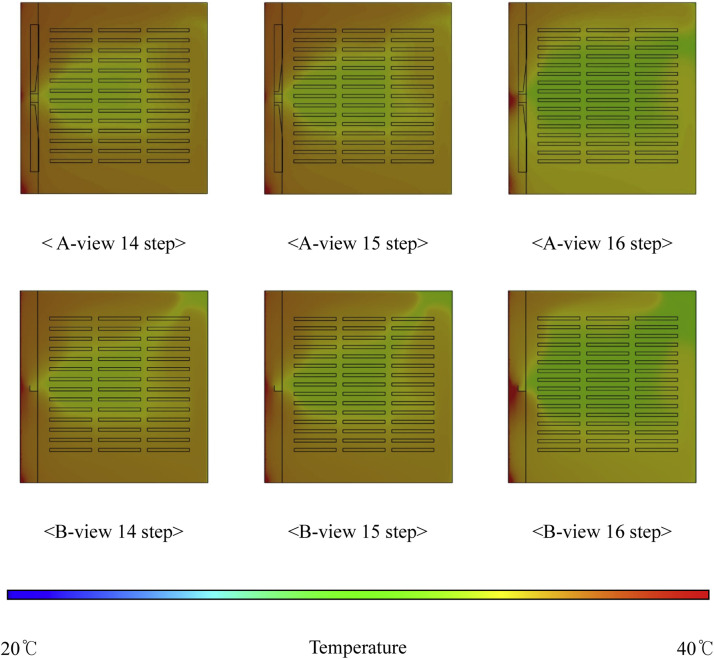
Contours of the internal temperature of the setter according to egg tray levels (based on 80 RPM, 0° tray angle).

**Fig. 19:**
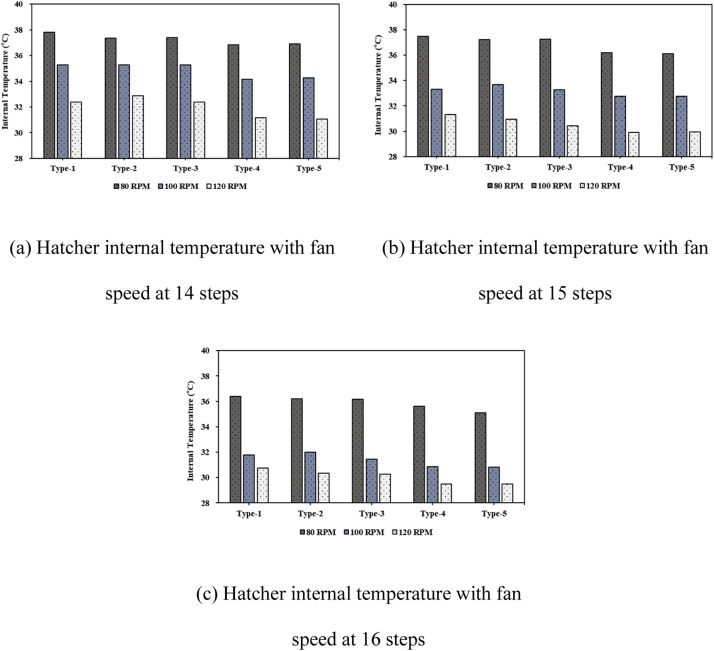
Internal temperature distribution in the setter according to the number of egg tray levels.

The internal thermal analysis results for setters based on the number of tray levels are shown in Fig. 18. This figure illustrates the simulation results corresponding to a fan speed of 80 RPM and an egg-tray turning angle of 0°. As the number of tray levels increased from 14 to 15 and 16, the average internal temperature of the setter decreased to 37.81 °C, 37.46 °C, and 37.12 °C, respectively. Additionally, the internal temperature distribution within the setter was non-uniform (Fig. 19, Table 8).

**Table 8:** Results of the heat flow analysis inside the egg setter (°C).

Type	14 levels			15 levels			16 levels		
	80 RPM	100 RPM	120 RPM	80 RPM	100 RPM	120 RPM	80 RPM	100 RPM	120 RPM
1	37.81	35.27	32.38	37.46	33.29	31.30	37.12	32.28	31.17
2	37.37	35.26	32.87	37.22	33.68	30.95	36.70	32.48	30.64
3	37.40	35.28	32.37	37.24	33.27	30.44	36.69	31.93	30.12
4	36.83	34.17	31.15	36.18	32.75	29.92	36.10	31.34	29.86
5	36.90	34.28	31.07	36.11	32.77	29.94	35.59	31.33	29.88

As the number of tray levels increased, the internal flow resistance within the setter increased, resulting in reduced internal air circulation. As air circulation decreased, the average internal temperature decreased, and temperature non-uniformity occurred.

In the study facility (KHS-576), the basic operation involved using 16 tray levels for hatching eggs. However, creating optimal conditions for air and thermal flow within the setter was challenging with 16 tray levels. Results from the thermal-fluid analysis showed that temperatures of 37.81 °C and 37.46 °C were maintained for optimal egg hatching when the egg trays were set to 14 and 15 tray levels, respectively, with an egg-tray turning angle of 0°. Considering improvements in egg productivity and economic efficiency, operating the setter with 15 tray levels was considered optimal and allowed for a greater number of eggs to be loaded.

**Fig. 20:**
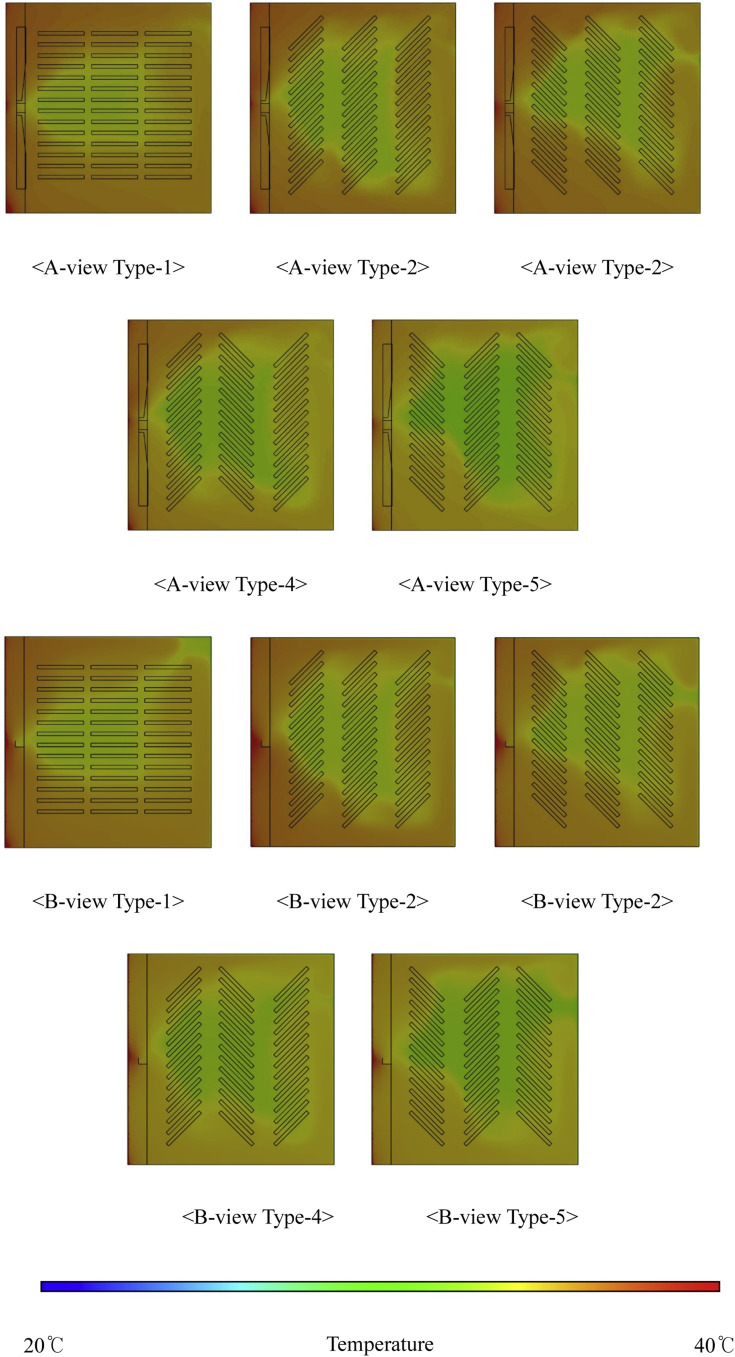
Contours of the internal temperature of the setter according to tray angle (based on 14 tray levels, 80 RPM).

**Fig. 21:**
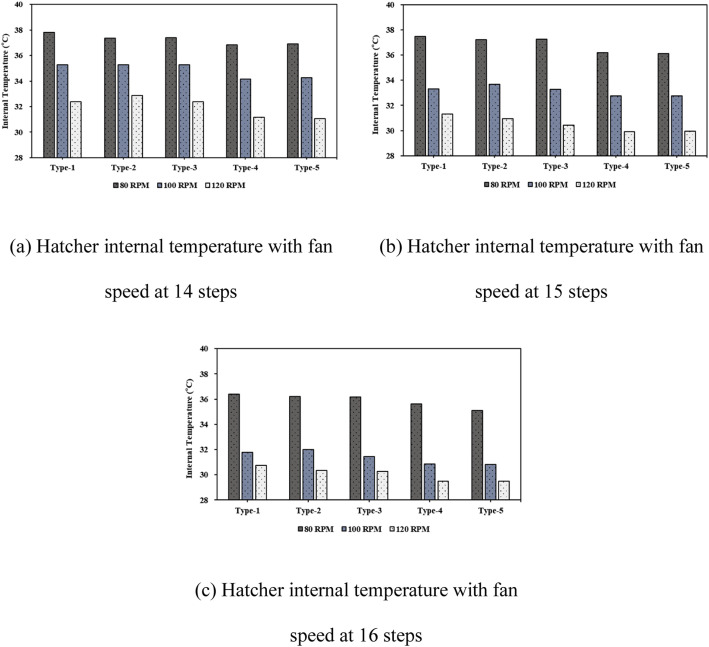
Internal temperature distribution in the setter according to the number of egg tray levels.

***Analysis of Internal Thermal Flow within the Setter according to Egg Tray Turning Angle.*** The thermal fluid analysis results obtained within the setter for different egg tray turning angles are presented in Fig. 20, which shows the temperature contours at 14 tray levels of the egg trays and a fan rotation speed of 80 RPM. Among the different types, Type-1 exhibited the most uniform temperature distribution within the setter and the highest average internal temperature. The temperatures were 37.81 °C for Type-1, 37.37 °C for Type-2, 37.40 °C for Type-3, 36.83 °C for Type-4, and 36.90 °C for Type-5. As observed from the temperature contours, the temperature distribution inside the setter was more uneven for bidirectional turning than for unidirectional turning. For unidirectional turning, the temperature was approximately 0.2–0.4 °C lower than at 0°, and for bidirectional turning, the internal temperature of the setter decreased even further, by approximately 1 °C compared to 0°. Additionally, increasing the fan speed under bidirectional turning compared to unidirectional turning led to a more uneven temperature distribution within the setter and a decrease in the average internal temperature (Fig. 21, Table 9).

**Table 9:** Results of the heat flow analysis inside the egg setter (°C).

Type	14 levels			15 levels			16 levels		
	80 RPM	100 RPM	120 RPM	80 RPM	100 RPM	120 RPM	80 RPM	100 RPM	120 RPM
1	37.81	35.27	32.38	37.46	33.29	31.30	37.12	32.28	31.17
2	37.37	35.26	32.87	37.22	33.68	30.95	36.70	32.48	30.64
3	37.40	35.28	32.37	37.24	33.27	30.44	36.69	31.93	30.12
4	36.83	34.17	31.15	36.18	32.75	29.92	36.10	31.34	29.86
5	36.90	34.28	31.07	36.11	32.77	29.94	35.59	31.33	29.88

This phenomenon is attributed to increased fluid resistance inside the setter and diminished air circulation caused by air impinging on the tray walls as the egg trays are turned to 45° and −45°. This leads to an uneven temperature distribution and a decrease in the overall temperature within the setter.

Egg tray turning is essential to prevent the adhesion of eggshell membranes to the inner tray surface and to enhance hatching productivity. When egg tray turning is implemented, it is recommended to use unidirectional egg turning, as in Types-2 and -3, to maintain optimal hatching temperatures.

### Analysis of internal humidity distribution within the hatcher under various operating conditions

This study analyzed the humidity distribution inside an egg setter using CFD simulations, focusing on a 15-level, 80 RPM condition. This configuration was selected because it provided the most stable and uniform internal temperature distribution in the previous analysis, making it an ideal basis for the humidity analysis.

The results showed that as the fan speed increased from 80 to 120 RPM, the humidity increased for all types. For instance, at with 14 tray levels, Type-1 recorded 70.86 % humidity at 80 RPM, which increased to 73.76 % at 120 RPM. Types-4 and -5 exhibited even more significant increases, with Type-5’s humidity rising from 74.06 % at 80 RPM to 77.13 % at 120 RPM. This indicates that an increased fan speed improves air circulation, leading to a more even humidity distribution within the setter, which is crucial for maintaining optimal hatching conditions.

As the number of tray levels increased, the humidity generally increased; however, the magnitude of the increase was modest. For example, at 14 tray levels, Type-1′s humidity increased from 70.86 % at 80 RPM to 73.76 % at 120 RPM, whereas Type-5 exhibited a similar increase from 74.06 % to 77.13 %. This suggests that while more tray levels improve the humidity distribution, the increase is limited owing to the higher air resistance, especially at higher RPMs.

The tray angle is also a key factor in humidity distribution. When the trays were aligned at 0°, humidity was more evenly distributed. However, inclined trays (at 45° and -45°) caused airflow disruption, leading to reduced humidity in some areas, particularly Types-2 and -3. This highlights the importance of the tray alignment in maintaining a uniform humidity distribution, even at higher fan speeds.

**Fig. 22:**
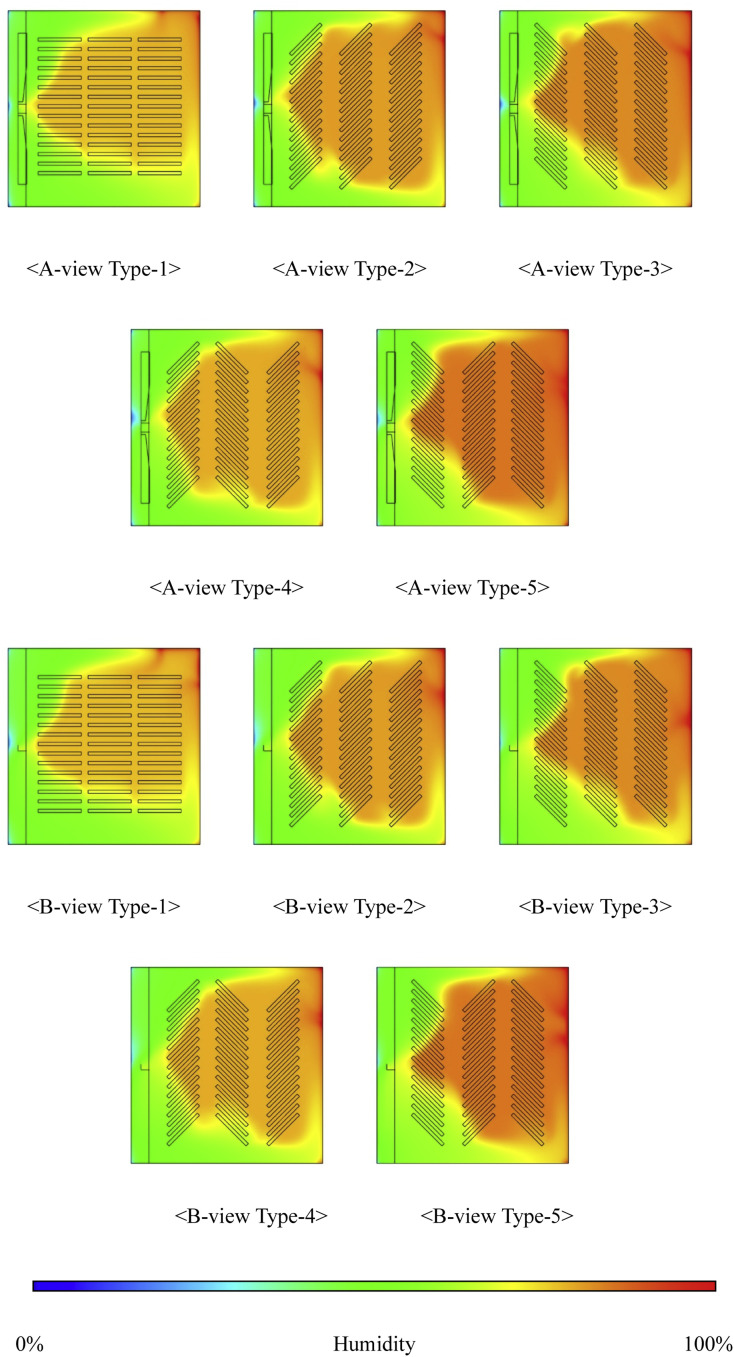
Contours of the internal humidity distribution of the setter according to tray angle (based on 15 tray levels, 80 RPM).

**Fig. 23:**
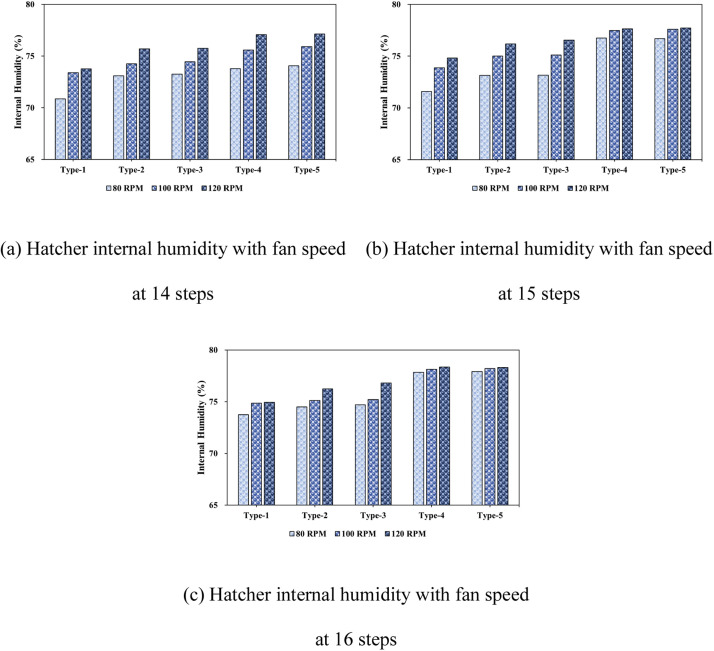
Internal humidity in the setter according to the number of egg tray levels.

Figs. 22 and 23 focus on the 15-level, 80 RPM condition, chosen for its optimal temperature stability. This condition was used to isolate the effects of fan speed and tray-level count on the humidity while maintaining a stable environment. The results confirm that this configuration provides the most uniform and stable humidity distribution, thereby creating ideal hatching conditions.

Additionally, Table 10 reinforces these findings, providing further data supporting the conclusion that 15 tray levels and 80 RPM yield the most consistent humidity levels inside the setter.

**Table 10:** Humidity measured inside the egg setter (%).

Type	14 levels			15 levels			16 levels		
	80 RPM	100 RPM	120 RPM	80 RPM	100 RPM	120 RPM	80 RPM	100 RPM	120 RPM
1	70.86	73.40	73.76	71.57	73.87	74.83	73.74	74.86	74.95
2	73.09	74.25	75.70	73.15	75.01	76.18	74.50	75.12	76.25
3	73.26	74.45	75.75	73.16	75.11	76.55	74.70	75.21	76.81
4	73.79	75.58	77.08	76.75	77.48	77.64	77.85	78.13	78.35
5	74.06	75.91	77.13	76.70	77.60	77.72	77.92	78.21	78.31

The patch feature was used to model the dynamic behavior of the humidification system inside the setter. Because the actual system maintained 70 % humidity by activating humidification when the level dropped below 70 %, the same mechanism was replicated in the CFD model. By initializing the internal humidity to 70 % using the patch feature, the simulation accurately reflected the real-world conditions of the setter, allowing for a realistic assessment of how fan speed, number of tray levels, and tray angle affect humidity distribution.

In conclusion, the 15-level, 80 RPM configuration ensures the most uniform humidity distribution, which is crucial for optimizing hatching conditions. This analysis underscores the need for careful optimization of the fan speed, number of tray levels, and tray angle to improve hatchability and overall egg productivity. Furthermore, maintaining appropriate humidity levels through dynamic regulation is essential for achieving high hatchability rates and improving egg development.

## Conclusions

In this study, CFD was used to analyze the internal airflow and thermal environment of large-scale setters under various operating conditions (fan speed, tray-level configuration, and egg tray spacing). The results of this study can be summarized as follows:

1) Airflow analysis showed that an increase in fan speed resulted in a higher internal air velocity within the setter, whereas an increase in the number of tray levels led to a decrease in air velocity, which affected the efficiency of airflow circulation.
2) Thermal flow analysis indicated that maintaining a fan speed of 80 RPM helped achieve uniform temperature distribution within the setter and maintains optimal hatching temperature conditions.
3) A 15-level egg tray configuration was found to be optimal for improving hatchability, and unidirectional egg turning was more effective in maintaining uniform temperature and humidity distributions.

These findings suggest that fan speed optimization and tray-level configuration play a critical role in enhancing the operational efficiency of large-scale setters by improving temperature uniformity and air circulation efficiency. However, it is important to note that the results of this study are specific to the system analyzed, and there may be differences when applied to other systems. In particular, this study did not consider differences in egg fertility rates, or variations in hatchery management practices, which could also influence setter performance. These factors represent important limitations that should be addressed in future work. Nonetheless, the trends identified in this study provide valuable foundational data for the optimization of large-scale setters. Specifically, efficient management of temperature and humidity by adjusting the tray-level configuration and fan speed plays a pivotal role in enhancing hatchability, which is essential for establishing standardized operational conditions in the future. Future studies should further investigate these additional factors and validate the findings across different systems to broaden their applicability. The results of this study therefore offer valuable insights into optimizing large-scale setter performance, thereby improving poultry production efficiency and sustainability. While some functions are already automated in commercial setters, our findings provide a validated, quantitative operating envelope that explicitly links operator-controllable parameters (fan speed, tray-level configuration, turning sequence) to measurable performance outcomes. This contribution goes beyond descriptive automation and can inform the refinement of automatic control rule-sets.

In addition, while hardware geometries such as tray, fan, and nozzle design may vary across manufacturers, the governing physical mechanisms can be framed using dimensionless groups (e.g., Reynolds, Rayleigh/Grashof, Sherwood numbers) and internal heat-source distribution. Thus, the operating trade-offs identified here (air velocity, temperature uniformity, humidity distribution) are expected to remain valid beyond the studied system. Future research will extend this work by exploring alternative tray/fan/nozzle geometries and conducting sensitivity analyses (e.g., fan diameter, nozzle placement variants), together with broader non-dimensional evaluations, to further assess the transferability of the proposed operating envelope.

## Funding

This work was supported by the IITP(Institute of Information & Communications Technology Planning & Evaluation)-ITRC(Information Technology Research Center) grant funded by the Korea government(Ministry of Science and ICT)(IITP-2025-RS-2024-00438335). This work was supported by Korea Institute of Planning and Evaluation for Technology in Food, Agriculture and Forestry (IPET) through Development of Smart Egg-hatchery with Air-conditioning System and ICT-based Realtime Control System Program, funded by Ministry of Agriculture, Food and Rural Affairs (MAFRA) (project no. RS-2021-IP821052). This work was supported by the research grant of Kongju National University, South Korea in 2024.

## CRediT authorship contribution statement

**Hee-woong Seok:** Writing – review & editing, Writing – original draft, Visualization, Validation, Project administration, Methodology, Investigation, Formal analysis, Data curation, Conceptualization. **Rack-woo Kim:** Writing – review & editing, Supervision, Resources, Project administration, Methodology, Funding acquisition. **Chan-min Kim:** Writing – review & editing, Writing – original draft, Visualization, Validation, Methodology, Investigation, Formal analysis, Data curation, Conceptualization. **Jun-gyu Kim:** Supervision, Resources.

## Disclosures

The authors declare that they have no known competing financial interests or personal relationships that could have appeared to influence the work reported in this paper.
